# Supplementary material for: Ethylene-Cytokinin Interaction Determines Early Defense Response of Wheat against Stagonospora nodorum Berk
Source: Biomolecules. 2021 Jan 28;11(2):174. doi: 10.3390/biom11020174 (PMC7911247; doi:10.3390/biom11020174)
Supplement: Supplementary file 1 [file biomolecules-11-00174-s001.pdf]

Supplementary Materials

Table S1. Primers used for qPCR.

Genes	Forward primers (5'→3')	Reverse primers (5'→3')
TaACS1	gag gtt gtg aac gag cta gaa g	ctc gaa acc ctg caa gag ata g
TaACO	tgt cca tcg cct cct tct a	cga aca cga acc ttg ggt at
TaEIL1	ggc tca aca acc tgg att tt	ccc tgt cca aag aaa ccc t
TaPIE1	aca cat gcc gac gaa tct ac	ccg agt tca aac cca aat tca c
TRbohD	acc acc aga cca gac aga c	tgg ttg gat agg agg cgt ag
TaRbohF	tgg cac ccc ttc tca att ac	ctc tcg tgt cca gtc acc aa
Ta PR1-4	ata acc tcg gcg tct tca tc	gct tat tac ggc att cct ttt
Ta PR2	gcg tga agg tgg tga ttt	gtg ccc gtt aca ctt gga t
TaIPT2	att cgt aca tgg gtg ttc cta tac	aat tgt tgg tac gta ggt gga g
TaIPT5	cga act agc tgt cat gcc ata g	aga gct tga ccg tag cag ta
TaZOG2-1	cct gca tgt cca agg agt tcc tc	gaa gga cac gta gag cac cga ct
TaGLU1-1	aat aac aac gca acg gtg aca gtt a	gat ccc gac gac atg caa aca
TaGLI4	acg tga cgg tcg acg agt acc ata	tct cca aac acc ttg aag cag aac t
TaCKX1	gga ggt ggc gct gga caa gat c	gca gaa ccg cag tat ctt ctg gt
TaRR21	tca acc agt cag cct ttc at	tag agc cca ttc cca tag ag
TaWRKY13	gag gag gaa cat gag cag aag	gaa cca ccc gaa gtc gaa g
TaRLI	ttg-agc-aac-tca-tgg-acc-ag	gct-ttc-caa-ggc-aca-aac-at

Table S2. The lists of orthologous genes used in this work.

Genes	Accession numbers	Orthologs	Accession numbers	References
TaACS1	U35779	*OsACS6*	AK065212	Subramaniam et al., 1996
TaACO	KF900072	*AtACO*	NM_127517	Zhang et al., 2019
TaEIL1	KU030837	*AtEIN3*	NM_112968	Duan et al., 2013
TaPIE1	EF583940	*AtERF1*	NM_113225	Zhu et al., 2014
TRbohD	AK335454	*AtRbohD*	NM_124165	Dmochowska-Boguta et al., 2013
TaRbohF	AY561153	*AtRbohF*	NM_105079	Dmochowska-Boguta et al., 2013
TaPR1-4	KF196278	*AtPR1*	NM_127025	Lu et al., 2011
TaPR2	DQ090946	*AtBGL2*	NM_115586	Lu et al., 2011
TaIPT2	JN128577	*ZmIPT2*	ABY78882	Song et al., 2012
TaIPT5	JN128579	*ZmIPT5*	NP001121194	Song et al., 2012
TaZOG2-1	JN128594	*ZmcZOG2*	Q8RXA5	Song et al., 2012
TaGLU1-1	JN128599	*ZmGLU2*	ACG24271	Song et al., 2012
TaGLU4	JN128604	*HvGLU*	ACF07988	Song et al., 2012
TaCKX1	JN128583	*ZmCKX1*	NP001105591	Song et al., 2012
TaRR21	JF951927	*AtRR2*	NM117704	Zhang et al., 2012
TaWRKY13	EF397614	*AtWRKY70*	NM_115498	Wu et al., 2008

Dmochowska-Boguta M., Nadolska-Orczyk A., Orczyk W. Roles of peroxidases and NADPH oxidases in the oxidative response of wheat (Triticum aestivum) to brown rust (Puccinia triticina) infection // Plant Pathology. 2013. 62, 993–1002. doi: 10.1111/ppa.12009.

Duan X., Wang X., Fu Y., Tang Ch., Li X., Cheng Y., Feng H., Huang L., Kang Zh. TaEIL1, a wheat homologue of AtEIN3, acts as a negative regulator in the wheat–stripe rust fungus interaction // Mol Plant Pathol. 2013. 14(7): 728–739. doi: 10.1111/mpp.12044.

Lu Sh., Friesen T.L., Faris J.D. Molecular characterization and genomic mapping of the pathogenesis-related protein 1 (PR-1) gene family in hexaploid wheat (Triticum aestivum L.) // Molecular Genetics and Genomics. 2011. 285.

Song J., Jiang L., Jameson P.E. Co-ordinate regulation of cytokinin gene family members during flag leaf and reproductive development in wheat // BMC Plant Biol. 2012. 6; 12:78. doi: 10.1186/1471-2229-12-78.

Subramaniam K., Abbo Sh. and Ueng P. P. Isolation of two differentially expressed wheat ACC synthase cDNAs and the characterization of one of their genes with root-predominant expression // Plant Molecular Biology 1996. 31. 1009-1020. doi: 10.1007/bf00040719.

Wu H., Ni Zh., Yao Y., Guo G., Sun Q. Cloning and expression profiles of 15 genes encoding WRKY transcription factor in wheat (Triticum aestivem L.) // Progress in Natural Science. 2008. 18(6). doi:10.1016/j.pnsc.2007.12.006.

Zhang, L., Zhao G., Jia J., Liu X. and Kong X. Molecular characterization of 60 isolated wheat MYB genes and analysis of their expression during abiotic stress // J. Exp. Bot. 63 (1), 203-214 (2012). doi: 10.1093/jxb/err264.

Zhang, S., Gan, Y. & Xu, B. Mechanisms of the IAA and ACC-deaminase producing strain ofTrichoderma longibrachiatum T6 in enhancing wheat seedling tolerance to NaClstress. BMCPlant Biol 19, 22 (2019). https://doi.org/10.1186/s12870-018-1618-5

Zhu X., Qi L., Liu X., Cai Sh., Xu H., Huang R., Li J., Wei X., Zhang Z. The Wheat Ethylene Response Factor Transcription Factor PATHOGEN-INDUCED ERF1 Mediates Host Responses to Both the Necrotrophic Pathogen Rhizoctonia cerealis and Freezing Stresses // Plant Physiol. 2014. 164(3): 1499–1514. doi: 10.1104/pp.113.229575.
